# Gut microbiome dynamics and associations with mortality in critically ill patients

**DOI:** 10.1186/s13099-023-00567-8

**Published:** 2023-12-19

**Authors:** Tarik J. Salameh, Katharine Roth, Lisa Schultz, Zhexi Ma, Anthony S. Bonavia, James R. Broach, Bin Hu, Judie A. Howrylak

**Affiliations:** 1 Division of Pulmonary and Critical Care Medicine, Milton S. Hershey Medical Center, Hershey, Penn State, PA 17033 USA; 2 Los Alamos National Laboratory, Los Alamos, USA; 3 Department of Anesthesiology and Perioperative Medicine, Penn State Milton S. Hershey Medical Center, Hershey, PA 17033 USA; 4 Institute for Personalized Medicine, Penn State College of Medicine, Hershey, PA 17033 USA; 5 Department of Biochemistry and Molecular Biology, Penn State College of Medicine, 500 University Drive, Hershey, PA 17033 USA

**Keywords:** Gut microbiome, ICU mortality, Predictive modeling

## Abstract

**Background:**

Critical illness and care within the intensive care unit (ICU) leads to profound changes in the composition of the gut microbiome. The impact of such changes on the patients and their subsequent disease course remains uncertain. We hypothesized that specific changes in the gut microbiome would be more harmful than others, leading to increased mortality in critically ill patients.

**Methods:**

This was a prospective cohort study of critically ill adults in the ICU. We obtained rectal swabs from 52 patients and assessed the composition the gut microbiome using 16 S rRNA gene sequencing. We followed patients throughout their ICU course and evaluated their mortality rate at 28 days following admission to the ICU. We used selbal, a machine learning method, to identify the balance of microbial taxa most closely associated with 28-day mortality.

**Results:**

We found that a proportional ratio of four taxa could be used to distinguish patients with a higher risk of mortality from patients with a lower risk of mortality (p = .02). We named this binarized ratio our microbiome mortality index (MMI). Patients with a high MMI had a higher 28-day mortality compared to those with a low MMI (hazard ratio, 2.2, 95% confidence interval 1.1–4.3), and remained significant after adjustment for other ICU mortality predictors, including the presence of the acute respiratory distress syndrome (ARDS) and the Acute Physiology and Chronic Health Evaluation (APACHE II) score (hazard ratio, 2.5, 95% confidence interval 1.4–4.7). High mortality was driven by taxa from the *Anaerococcus* (genus) and *Enterobacteriaceae* (family), while lower mortality was driven by *Parasutterella* and *Campylobacter* (genera).

**Conclusions:**

Dysbiosis in the gut of critically ill patients is an independent risk factor for increased mortality at 28 days after adjustment for clinically significant confounders. Gut dysbiosis may represent a potential therapeutic target for future ICU interventions.

## Introduction

The human colon boasts the richest density of life on record with more than a trillion organisms per milliliter [1], housing a rich ecosystem that plays crucial roles in regulating immune response [2] and defense against infection [3]. Thus, it is not surprising that gut dysbiosis is associated with sepsis, a critical illness driven by dysregulated immune response to infection. Evidence that dysbiosis drives sepsis and other critical illnesses has been apparent in animal studies over the last decade [4, 5]. Evidence in humans is similarly compelling. Gut dysbiosis has been implicated as a major risk factor for sepsis in a large epidemiological study [6], and among patients admitted to intensive care units (ICUs), dysbiosis is strongly associated with mortality, infection, and length of hospital stay [7–15].

In healthy adults, there is a large amount of diversity in the community composition of the gut microbiome [1]. However, several studies have shown that critical illness leads to rapid dysbiosis and loss of diversity [8, 9, 11–14]. Further evidence suggests that changes in taxa, such as an increase in *Enterococcus* [7, 15], or an increase in the ratio of *Bacteroidetes* to *Firmicutes* [10] may be associated with increases in organ failure and ICU mortality. However, further studies are necessary to characterize the impact of changes in the composition of the human gut on the course of critical illness.

It is in this context that we present the following analysis with two aims: (1) quantify the effect gut microbiota variation has on ICU outcomes and (2) identify specific gut microbiota associated with adverse outcomes. We performed 16 S RNA sequencing of stool samples from 52 critically-ill ICU patients who required ventilator support, and we document links between gut microbiota and 28-day mortality.

## Methods

### Patient population and specimen collection

Patients were enrolled in the PSHMC Critical Illness Registry (CIR). In the CIR, critically ill patients receiving mechanical ventilation were enrolled on a rolling basis within 48 h of admission to the Medical Intensive Care Unit (MICU) at PSHMC between 2013 and 2017. Clinical and demographic data from each subject’s admission was abstracted from the electronic medical record. Mortality was assessed at 28-days. Fecal specimens were obtained from rectal swabs of the anal vault on the date of enrollment, then stored at -80 degrees Celsius until the time of processing.

### Outcomes and variables

The primary outcome was 28-day mortality. The secondary outcome was the number of death-free days from admission to the MICU. Survival was monitored for one year from admission to the MICU. Variables abstracted from patient records are listed in Table 1. Categorical variables indicate presence or absence on the day of specimen collection.

**Table 1:** Clinical and demographic characteristics of study subjects

Variables	Total patients (N = 52) (Median [IQR] or n (%))
Age	67 [22]
Sex (Male)	24 (46%)
Race (Caucasian)	49 (94%)
APACHE II score	27 [11]
PF ratio	156 [103]
Comorbidities	
ARDS Sepsis-3 Septic Shock Malignancy Diabetes Immunosuppressed COPD	26 (50%) 34 (65%) 28 (54%) 13 (25%) 18 (35%) 20 (38%) 7 (13%)
Diet	
Not being fed Enteral diet Oral diet	28 (53.8%) 21 (40.4%) 3 (5.8%)
Antibiotics	
Azithromycin Cefepime	9 (17%) 10 (19%)
Ceftriaxone Ciprofloxacin Piperacillin-tazobactam Vancomycin	14 (27%) 12 (23%) 31 (60%) 28 (54%)
Outcomes	
Death < 1 year Death < 28 days Death-free days	38 (73%) 28 (54%) 24 [354]

Cohort clinical characteristics as measured on first day of admission to the ICU. Antimicrobial variables represent whether or not a given drug was administered on the day of specimen collection. APACHE: acute physiology and chronic health evaluation. PF ratio: ratio of partial pressure of oxygen to fraction of inspired oxygen. Sepsis-3: ARDS: acute respiratory distress syndrome. Sepsis-3: Third International Consensus definition of sepsis. COPD: chronic obstructive pulmonary disease. IQR: Interquartile range

### Specimen processing and sequencing

Specimens were sequenced as previously described [16], then sequenced on an Illumina MiSeq. 16 S RNA sequences were processed using dada2 via QIIME2 [17, 18]. Sequences were assigned to taxonomies using IDTaxa at a confidence score of 80, equivalent to an error rate < 1% [19].

### Identifying taxa associated with increased mortality

To identify a microbe signature associated with mortality in critically ill patients, a microbiome mortality index (MMI), we employed selbal, a supervised learning framework that searches for microbial balances [20]. “Balance” here refers to the log ratio between geometric means of two subsets of taxa (Eq. 1); intuitively, between-taxa ratios within a specimen should mirror those within its host. Selbal performs two steps: (1) determine the optimal number of taxa in a balance then (2) search for a balance that improves the predictive performance of a logistic regression model. Selbal returns the simplest balance that has predictive ability comparable to the best-performing balance during cross-validation. Our target variable was 28-day mortality. We refer readers to Rivera-Pinto et al. for further details on selbal’s algorithm [20].

Taxa were counted at the genus level; the *Enterobacteriaceae* family was also included due to limited differentiation of its genera by the selected 16 S regions [21]. Taxa unobserved in at least 20% of specimens were excluded. Models trained by selbal included age and APACHE II scores as covariates. We used default settings except for two adjustments. First, we replaced zeros with random values between 0.01 and 1 as this better preserved correlation structures when sparsity was high [22]. Second, we reported balances calculated using base ten logs instead of the natural base to improve interpretability. Please see Supplementary Figs. 1–4 for further details regarding selbal parameter tuning in our patient cohort. We developed two balances: one associated with high mortality (high MMI) and one associated with comparatively lower mortality (low MMI).

### Statistical analysis

We analyzed differences in microbial ecology between patients in high and low MMI groups using the vegan package 2.6-4 and mvabund 4.2.1 in R [23–25]. We compared microbial community diversity (alpha diversity) using metrics for evenness (Pielou, Simpson), diversity (Shannon, Simpson, Fisher), richness (Chao1), and dominance [26]. For evenness, higher values indicated more equal abundances among species within a sample. The different diversity metrics represent different forms of weighted means between species; for example, Shannon is a weighted geometric mean of proportional abundances in log2 scale. Richness estimates the total number of species present in a sample. Dominance is the inverse of evenness, thus high dominance indicates low diversity. We also evaluated differences in microbial community composition (beta diversity) using rank abundance analysis.

We used Wilcoxon rank sums tests to assess stochastic equality of quantitative variables in patients grouped by 28-day mortality; We used Fisher’s exact test for categorical variables. And Student’s t tests to evaluate differences in means in patients grouped by binarized (positive or negative) MMI. We used sparse PCA on CLR-transformed taxa abundances to visualize trends between sparse components and MMI. We fit regularized (l1 ratio 0·5) Cox proportional hazards models using the first 28 days of each patients admission.

### Software

We used python v3.8.10 or R v4.2.0 for all analyses. For sequencing processing, we used Dada2 (1.18) within the QIIME2 framework (2021.11) [18]. We used IDTaxa (2.20.0) for assigning taxonomies. We used Scikit-bio (0.5.6) for diversity metrics and CLR transforms. We fit Cox proportional hazards models using lifelines (0.26.4) in Python.

### Role of the funding source

Funding sources did not have a role in study design, data collection, analysis, interpretation, or manuscript preparation.

## Results

### Cohort characteristics

Demographics and clinical characteristics of patients are reported in Table 1. Cohort demographics reflect the communities of central Pennsylvania, where the study was conducted. The median age was 67.4 [21.6], 53.8% (28 patients) of patients were male, and 94.2% (49) of patients were Caucasian.

The cohort had a high level of disease severity. The cohort’s median APACHE II score was 27 [11], 65% (34) of patients met sepsis-3 criteria, and 50% (26) of patients had ARDS. In this cohort, 38.5% (20) of patients were immune suppressed, most often from recent or current immune suppressive therapy, 34.6% (18) had diabetes, and 25% (13) had documented malignancy. Over one half (53.8%) of the cohort more than half of patients died within 28 days of enrollment, and nearly three quarters (73.1%) died within one year.

We tracked antimicrobial administration to patients within the study cohort. The most frequent antimicrobials administered were piperacillin-tazobactam (59.6%), followed by vancomycin (53.8%), ceftriaxone (26.9%), ciprofloxacin (23.1%), and cefepime (19.2%). We also tracked the diet of patients in our cohort, and found that a majority of patients were not being fed during the study (53.8%), most patients being fed were receiving an enteral diet (tube feeding) (40.4%). A small number of patients were receiving an oral diet (5.8%).

### Increased risk of ICU mortality is characterized by distinct microbial profiles

We found that different proportions of gut taxa could be used to distinguish between patients at higher vs. lower risk of 28-day mortality. This proportion, which we named the microbiome mortality index (MMI), consisted of a ratio between four bacterial taxa, with *Enterobacteriaceae* and *Anaerococcus* forming a “numerator” subset, and correlating positively with mortality, and *Parasutterella* and *Campylobacter* forming a “denominator” subset, and correlating negatively with mortality.

**Fig. 1:**
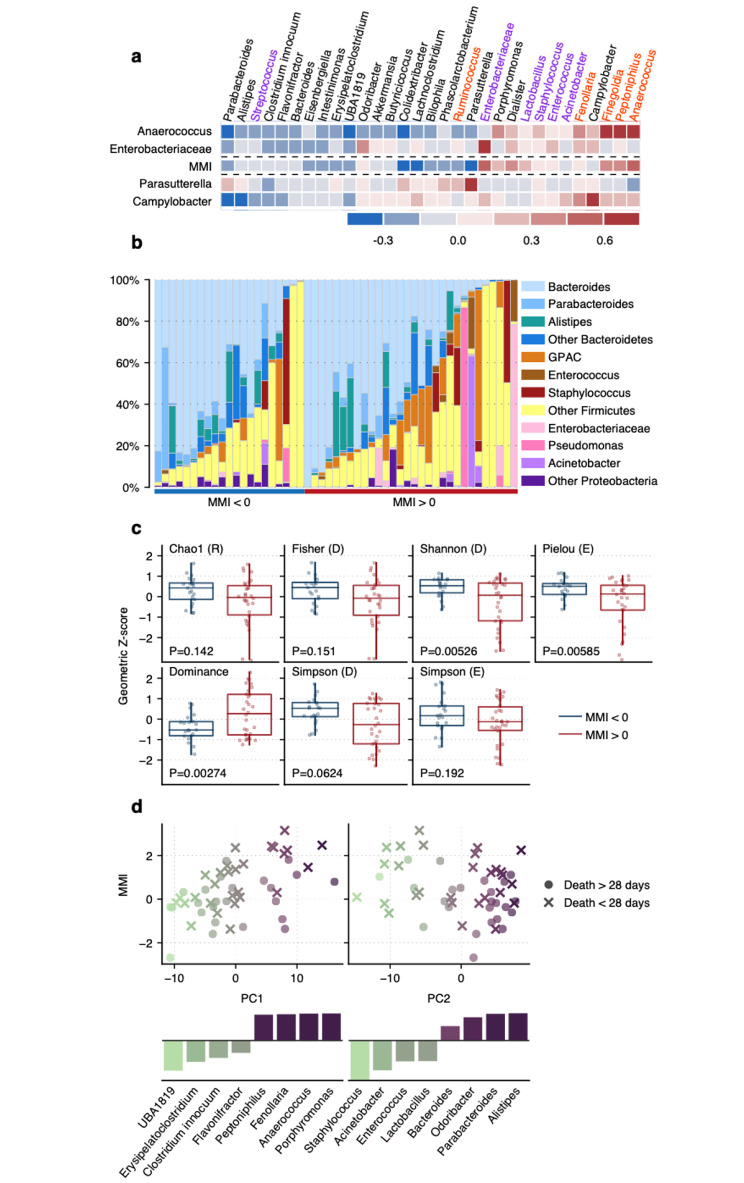
Characterization of the difference in microbial taxa between patients with higher mortality (MMI > 0) compared to patients with lower mortality (MMI < 0). **A**) Heatmap of Spearman correlations between 30 taxa (columns) and the four component taxa used to calculate MMI (rows). The top two rows are the taxa present in the numerator of the MMI ratio and the bottom two rows are the taxa present in the denominator of the MMI. The middle row (MMI) depicts overall correlations with the MMI. There is strong collinearity between *Anaerococcus* and other GPACs (columns in red print). *Parasutterella* correlates with commensals (columns in black print). In general, the MMI was positively correlated with GPACs as well as members of the *Enterobacteriaceae* family (purple print), but was negatively correlated with commensal organisms. **B**) Bar plot of observed relative abundances of taxa for each patient. Patients with higher MMIs were more frequently dominated by a single taxon, and also had higher relative abundance of GPACs. **C**) Alpha diversity box plots grouped by MMI binarized at zero. Diversity was lower among high MMI patients. “R” denotes richness, “D” denotes diversity, “E” denotes evenness. **D**) SSPCA showing each identified principal component plotted against MMI. Relative PC weights for associated taxa are shown as bar plots. Color used only to aid interpretation of each taxon’s direction of effect

Patients in the higher mortality subgroup (MMI > 0) were characterized by two qualitative patterns of gut microbiota disturbance; these were increased abundance of Gram Positive anaerobic cocci (GPACs) and increased abundance of oxygen-tolerant bacteria from the *Enterobacteriaceae* family (Fig. 1a and b). In these patients, *Anaerococcus* was positively correlated with other GPACs, such as *Peptoniphilus* and *Finegoldia*, and serves as a representative taxon for GPAC enrichment (Fig. 1a). Similarly, *Enterobacteriaceae* was positively correlated with oxygen-tolerant bacteria, including *Staphylococcus* and *Enterococcus* (Fig. 1a). Commensal taxa are those that live in symbiosis with their human host, and provide beneficial functions, such as production of nutrients, stimulation of the immune system, defense against colonization by opportunistic pathogens, and contribution to the development of the intestinal architecture [27]. Commensal organisms measured in our dataset included *Butyricicoccus*, *Alistipes, Akkermansia, Bilophilia, Dialister, and Clostridium innocuum.* We observed that both *Anaerococcus* and *Enterobacteriaceae* were negatively correlated with commensal taxa in the gut. Conversely, *Parasutterella* in the denominator of the MMI ratio was positively correlated with commensal organisms (Fig. 1a).

We found that measures of microbial diversity were altered in patients with a MMI > 0 compared to patients with a MMI < 0. Two major components to biodiversity are richness (the number of species in an ecosystem) and evenness (the extent to which species are evenly distributed in an ecosystem) [26]. There was a significant difference in evenness (as measured by Pielou, Simpson, and Dominance indices) between patients grouped by positive or negative MMI; patients with a MMI < 0 had higher richness and, to a greater extent, had higher evenness (Fig. 1c).

We performed a sparse supervised principal component analysis (SSPCA) on center-log transformed abundance levels. Our analysis yielded two component vectors, which we interpreted as quantitative patterns of gut microbiota disruption (Fig. 1d). One component vector was the ratio between GPACs and commensal taxa. The other component vector was the ratio between commensal *Bacteroidetes* are anaerobic bacteria. Both vectors correlated with MMI, suggesting that the MMI is a marker for specific patterns of gut microbiota disturbance found in the ICU.

### Both clinical and microbial variables are associated with increased mortality

We performed a bivariate analysis of death-free days as a function of multiple clinical and composite variables. We hypothesized that the joint effect of changes in microbial taxa held more clinical significance than changes in the relative abundance levels of individual taxa alone. For this reason, we used the MMI as a composite variable composed of the ratio of four specific taxa, as described in the previous section (Table 2). There was a statistically significant difference in 28-day mortality between patients with a positive MMI vs. a negative MMI (Fisher’s exact P = .023). The proportion of patients in our dataset with a positive MMI was 60% (31 patients), but these patients represented only 19% of survivors (10 patients). APACHE II scores were higher in patients who died within 28 days of MICU admission (median 31, IQR 9.2) than in patients who did not die (median 27, IQR 11) (Wilcoxon rank sum P = .012). There were no significant differences in 28-day mortality between patients who used specific antimicrobials or between different metrics of microbial diversity (Table 2).

**Table 2:** Bivariate analyses between observed variables and 28-day mortality

	Median[IQR] or n	Death < 28 days	Death > 28 days	P value
Sex (male)	24 (46%)	11 (21%)	13 (25%)	0.4
APACHE II score	27.0 [ 21.0, 32.2]	31.0 [25.8, 35.0]	24.5 [ 20.0, 28.8]	0.012
ARDS	26 (50%)	19 (37%)	7 (13%)	0.012
Malignancy	13 (25%)	10 (19%)	3 (6%)	0.064
Immunosuppressed	20 (38%)	13 (25%)	7 (13%)	0.26
Diabetes	18 (35%)	11 (21%)	7 (13%)	0.56
Sepsis-3	34 (65%)	19 (37%)	15 (29%)	0.77
Vancomycin	28 (54%)	18 (35%)	10 (19%)	0.16
Ceftriaxone	14 (27%)	6 (12%)	8 (15%)	0.37
Ciprofloxacin	12 (23%)	8 (15%)	4 (8%)	0.35
Piperacillin-tazobactam	31 (60%)	19 (37%)	12 (23%)	0.26
MMI	0.3 [-0.2, 1.3]	0.9 [0.0, 1.7]	-0.1 [-0.7, 0.6]	0.01
MMI > 0	31 (60%)	21 (40%)	10 (19%)	0.023
Shannon (D)	5.8 [ 4.7, 6.2]	5.8 [ 4.7, 6.2]	5.9 [ 4.7, 6.2]	1.0
Chao1 (R)	102.0 [ 76.5, 133.2]	102.0 [ 76.5, 127.5]	99.5 [ 77.8, 144.8]	0.71

### Gut microbiota are predictive of 28-day mortality in ICU patients

**Fig. 2:**
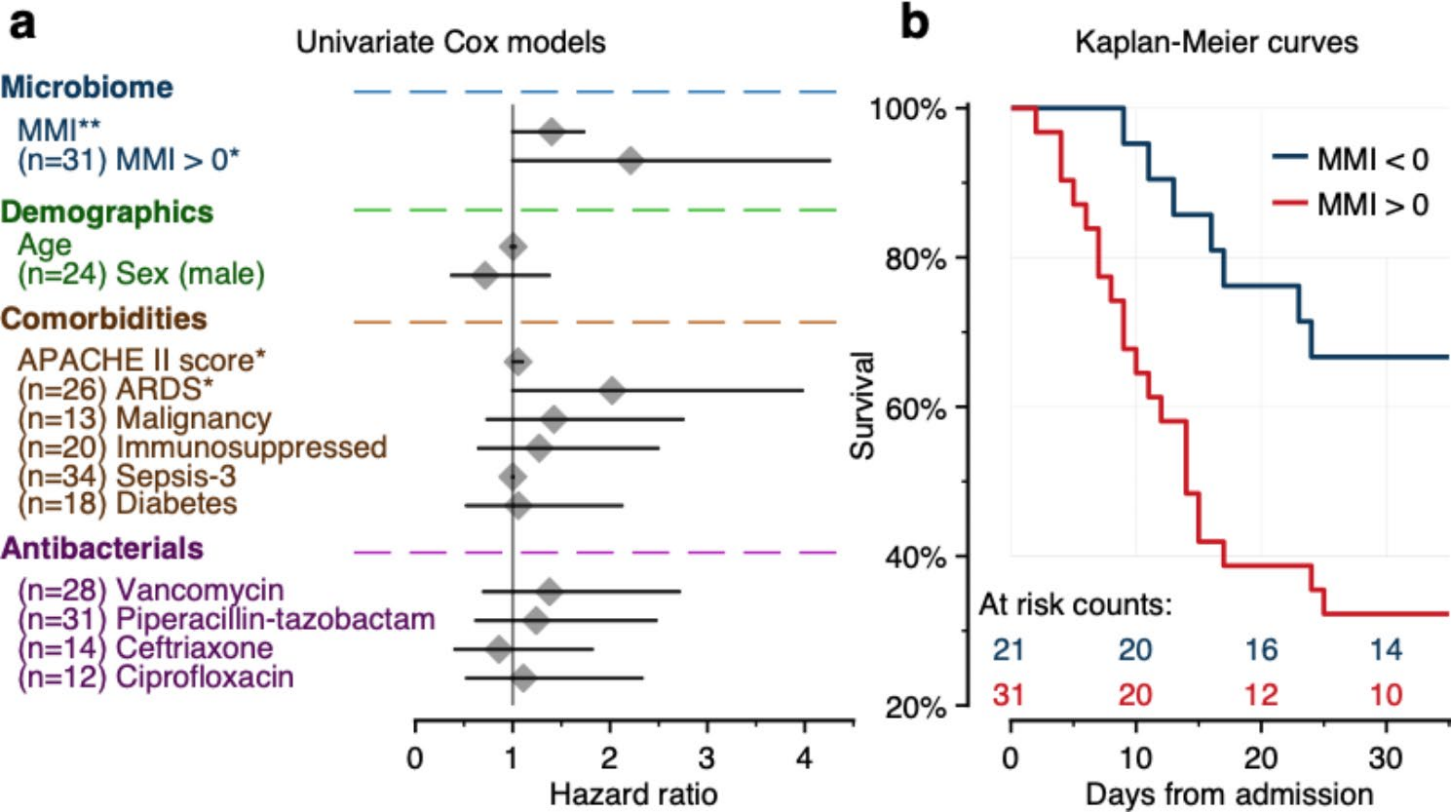
Gut microbial taxa are associated with mortality in critically ill patients. Results of **(A)** 95% confidence intervals for Hazard Ratio (HR) in univariate Cox models using clinical variables and microbial taxa in the form of the MMI as predictors of 28-day mortality. MMI, binarized MMI, APACHE II score, and ARDS all had a lower bound that was > 1. **(B)** Kaplan–Meier survival curves for patients grouped by binarized MMI. 10 of 31 high MMI patients survived by day 30, compared to 14 of the survivors in the low MMI group

We used regularized univariate Cox proportional hazards models to assess the relationship between multiple clinical variables and the number of death-free days in our cohort (Table 3; Fig. 2a). We used the MMI as a measure of gut microbiome dynamics and used a continuous version of the variable, as well as a binarized version of the variable (with values of > or < 0). In univariate analysis, we found significant associations between 28-day mortality and APACHE II score, the diagnosis of ARDS, and both MMI and MMI binarized at 0. In multivariate models, a combination of MMI or binarized MMI with clinical variables had better predictive ability than multivariate models using only clinical variables (Table 3). Importantly, APACHE II score and ARDS remained significant and usually increased in significance when MMI was a covariate, suggesting that MMI was capturing an independent component of mortality variation. Kaplan–Meier analysis of patients grouped by binarized MMI was concordant with Cox modeling. Among 31 patients with MMI > 0, 21 died within 28 days, compared to seven of 21 patients with MMI < 0 (Fig. 2b).

**Table 3:** Cox Proportional Hazards Analysis

Univariate Models	Variable	Hazard ratio (95% CI)	P value
Age	Age	1 (0.98, 1)	0.67
Sex (male)	Sex (male)	0.72 (0.37, 1.4)	0.32
Diabetes	Diabetes	1.1 (0.53, 2.1)	0.87
Malignancy	Malignancy	1.4 (0.74, 2.7)	0.29
Immunosuppressed	Immunosuppressed	1.3 (0.65, 2.5)	0.48
Sepsis-3	Sepsis-3	1 (1, 1)	0.84
ARDS	ARDS	2 (1, 4)	0.041
APACHE II score	APACHE II score	1.1 (1, 1.1)	0.01
MMI > 0	MMI > 0	2.2 (1.1, 4.3)	0.017
**Bivariate Models**			
(APACHE II score) + ARDS	ARDS	1.8 (1.0, 3.5)	0.066
(APACHE II score) + ARDS	APACHE II score	1.1 (1.0, 1.1)	0.034
(APACHE II score) + MMI > 0	APACHE II score	1.1 (1.0, 1.1)	0.0033
(APACHE II score) + MMI > 0	MMI > 0	2.5 (1.4, 4.6)	0.0029
ARDS + (MMI > 0)	MMI > 0	2.3 (1.2, 4.2)	0.01
ARDS + (MMI > 0)	ARDS	2.1 (1.1, 4.1)	0.035
**Multivariate Models (3 variables)**			
(APACHE II score) + ARDS + (MMI > 0)	APACHE II score	1.1 (1.0, 1.1)	0.0094
(APACHE II score) + ARDS + (MMI > 0)	ARDS	1.9 (1.0, 3.6)	0.062
(APACHE II score) + ARDS + (MMI > 0)	MMI > 0	2.5 (1.4, 4.7)	0.0026

## Discussion

One of the most notable findings to arise from this analysis was the importance of the ratios between specific bacterial taxa in predicting mortality risk. Previous studies of the gut microbiome in critical illness showed both a loss of bacterial diversity [2–13, 27] and a rise in the presence of pathologic bacteria [7, 11, 12, 15] among critically ill patients. One recent study of twelve patients found that differences in relative abundance levels of *Bacteroidetes* and *Firmicutes* phyla corresponded to differences in subsequent mortality [10]. Similar to earlier studies, the current study confirmed that patients with increased mortality had a lower level of bacterial diversity and increased levels of pathobionts. However, the current goes a step further by establishing the importance of ratios between pathobionts and commensal organisms in the gut in patients with increased mortality.

In developing our MMI, we found that an increased risk of mortality was characterized by an increase in GPAC and *Enterobacteriaceae* at the expense of commensal taxa. GPACs are anaerobic cocci that typically colonize the skin and mucosal surface of the human body [28]. When patients become immunocompromised, or develop skin wounds, GPACs can cause invasive infections [29]. The presence of GPACs as a key component of our MMI predictor suggests that invasion of these organisms is associated with mortality in critically ill patients. Gram negative rods, such as the *Enterobacteriaceae* present as a component of our MMI, are well-known pathobionts in critically ill ICU patients, present in many nosocomial infections [30]. For our analysis, we grouped these organisms as a family because we found that taxa within the *Enterobacteriaceae* family could not be reliably distinguished from one another in our dataset. *Enterobacteriaceae* infections are significant in critically ill patients because they are associated with widespread antimicrobial resistance [31].

Components of the MMI that were inversely associated with mortality were the *Parasutterella* and *Campylobacter* taxa. *Parasutterella* is a gram negative anaerobic organism, and is understood to be a core member of the healthy gut microbiome [32]. The presence of *Parasutterella* in the denominator of the MMI suggests that decreased commensal organisms are associated with increased mortality in the ICU. The presence of *Campylobacter* in the denominator of the MMI is somewhat more difficult to explain. *Campylobacter* is a commensal organism in many animals, but is typically a mediator of food-borne illness in humans [32]. Its role in the MMI is unclear. One explanation for its role in the MMI involves its interaction with carbohydrate and amino acid metabolism, which are converted to SCFAs in states of symbiosis. *Campylobacter* was recently shown to have enhanced growth in the presence of *Bacteroides vulgatus*, possibly due to a scavenging interaction [33]. We observed a positive correlation between *Campylobacter* and butyrogenic genera such as *Odoribacter*, *Lachnoclostridium*, and *Butyricoccus*, as well as the protective *Akkermansia* genus and potentially butyrogenic *Colidextribacter*. Notably, *Parasutterella* had negative correlations with the latter two, suggesting that *Campylobacter* signals the presence of certain commensals that *Parasutterella* does not. In other words, if *Campylobacter* are abundant, something may be feeding them.

Another notable finding arising from this analysis with the effect of the MMI as a predictor of mortality. Consistent with earlier studies, we found that the presence of ARDS as well as a high APACHE II score were significant predictors of 28-day mortality in critically ill patients [34, 35]. However, we also found that MMI was a significant predictor of 28-day mortality and outperformed both ARDS and APACHE II, with a multivariate model containing MMI, ARDS, and APACHE II a particularly strong predictor of mortality. This result suggests that MMI and specifically disruption of homeostasis in the intestinal tract of critically ill patients is predictive of increased mortality. Implicit in this finding is that correction of this dysbiosis may lead to decreased mortality.

Our study had several limitations. First, the development of MMI was limited by our small sample size. We attempted to minimize the impact of small sample size in the development of our model using cross validation to minimize overfitting of the model to a small dataset. We were also limited by our lack of an independent validation cohort. This will need to be addressed in future studies.

In summary, we have developed the MMI, a biomarker based upon ratios of different taxa from the gut. The MMI is an accurate predictor of 28-day mortality that augments previous mortality predictors in the ICU, including the presence of ARDS and APACHE II score. Further studies will be necessary to confirm these findings, including confirmation in an independent validation cohort.

## Data Availability

All publicly unreleased gene expression data will be uploaded to the Gene Expression Omnibus (GEO) once accepted for publication. For the purpose of review, the complete dataset used for analysis is currently available via the following link: https://www.github.com/tariks/icu-biome.

## Declarations

### Take home message

Prior studies of the gut microbiome in critically ill patients have indicated that specific taxa may be associated with increased mortality. In the current analysis, we extend these earlier findings by identifying a gut microbiome biomarker associated with increased mortality, and demonstrating how this biomarker may be used to enhance predictive models using clinical data alone.
